# Loeys-Dietz syndrome type I and type II: clinical findings and novel mutations in two Italian patients

**DOI:** 10.1186/1750-1172-4-24

**Published:** 2009-11-02

**Authors:** Bruno Drera, Marco Ritelli, Nicoletta Zoppi, Anita Wischmeijer, Maria Gnoli, Rossella Fattori, Pier Giacomo Calzavara-Pinton, Sergio Barlati, Marina Colombi

**Affiliations:** 1 Division of Biology and Genetics, Department of Biomedical Sciences and Biotechnology, University of Brescia, Brescia, Italy; 2 Medical Genetics Unit, University Hospital S. Orsola-Malpighi, Bologna, Italy; 3 Cardiovascular Department, University Hospital S. Orsola-Malpighi, Bologna, Italy; 4 Division of Dermatology, University of Brescia and Azienda Ospedaliera Spedali Civili, Brescia, Italy

## Abstract

**Background:**

Loeys-Dietz syndrome (LDS) is a rare autosomal dominant disorder showing the involvement of cutaneous, cardiovascular, craniofacial, and skeletal systems. In particular, LDS patients show arterial tortuosity with widespread vascular aneurysm and dissection, and have a high risk of aortic dissection or rupture at an early age and at aortic diameters that ordinarily are not predictive of these events. Recently, LDS has been subdivided in LDS type I (LDSI) and type II (LDSII) on the basis of the presence or the absence of cranio-facial involvement, respectively. Furthermore, LDSII patients display at least two of the major signs of vascular Ehlers-Danlos syndrome. LDS is caused by mutations in the transforming growth factor (TGF) beta-receptor I (*TGFBR1*) and II (*TGFBR2*) genes. The aim of this study was the clinical and molecular characterization of two LDS patients.

**Methods:**

The exons and intronic flanking regions of *TGFBR1 *and *TGFBR2 *genes were amplified and sequence analysis was performed.

**Results:**

Patient 1 was a boy showing dysmorphic signs, blue sclerae, high-arched palate, bifid uvula; skeletal system involvement, joint hypermobility, velvety and translucent skin, aortic root dilatation, tortuosity and elongation of the carotid arteries. These signs are consistent with an LDSI phenotype. The sequencing analysis disclosed the novel *TGFBR1 *p.Asp351Gly *de novo *mutation falling in the kinase domain of the receptor. Patient 2 was an adult woman showing ascending aorta aneurysm, with vascular complications following surgery intervention. Velvety and translucent skin, venous varicosities and wrist dislocation were present. These signs are consistent with an LDSII phenotype. In this patient and in her daughter, *TGFBR2 *genotyping disclosed in the kinase domain of the protein the novel p.Ile510Ser missense mutation.

**Conclusion:**

We report two novel mutations in the *TGFBR1 *and *TGFBR2 *genes in two patients affected with LDS and showing marked phenotypic variability. Due to the difficulties in the clinical approach to a TGFBR-related disease, among patients with vascular involvement, with or without aortic root dilatation and LDS cardinal features, genotyping is mandatory to clarify the diagnosis, and to assess the management, prognosis, and counselling issues.

## Background

Loeys-Dietz syndrome (LDS) is a rare autosomal dominant disorder characterized by hypertelorism, bifid uvula and/or cleft palate, and arterial tortuosity with widespread vascular aneurysm and dissection [1]. LDS shows the involvement of cutaneous, cardiovascular, craniofacial and skeletal systems [1,2]. LDS patients have a high risk of aortic dissection or rupture at an early age and at aortic diameters that ordinarily are not predictive of these events [2]. Recently, LDS has been subdivided in LDS type I (LDSI) and type II (LDSII) on the basis of the presence or the absence of cranio-facial involvement, respectively [2]. Furthermore, LDSII patients display at least two of the major signs of vascular Ehlers-Danlos syndrome (vEDS) [2].

Mutations in the transforming growth factor (TGF) beta-receptor type I (*TGFBR1*) and type II (*TGFBR2*) genes have been found to cause LDS [1]. Mutations in these genes were also reported in other syndromes showing cardiovascular involvement, with or without marfanoid *habitus *or fulfilled Ghent criteria for Marfan syndrome (MFS) and variable association with signs characteristic of LDS: MFS type 2 (MFS2), Furlong syndrome (FS), familial thoracic aortic aneurysms and dissections type 2 (TAAD2), and Shprintzen-Goldberg syndrome, the nosological status of which is still controversial [3-9]. All these syndromes should be considered as a phenotypic *spectrum *associated with aberrant TGFbeta signalling [9].

TGFBR1 and TGFBR2 are transmembrane serine/threonine kinases, both able to bind TGFbeta. Ligand binding mediates TGFbeta signalling activation [10]. They are composed of a small cysteine-rich extracellular domain, a single transmembrane region, and an intracellular kinase domain [10]. So far, less than 30 *TGFBR1 *and less than 100 *TGFBR2 *disease causing mutations have been described [1-6,11-13].

Here we report the characterization of two Italian LDS patients and the identification of two novel mutations, one in the *TGFBR1 *and the other in the *TGFBR2 *gene.

## Case Presentation

**Figure 1:**
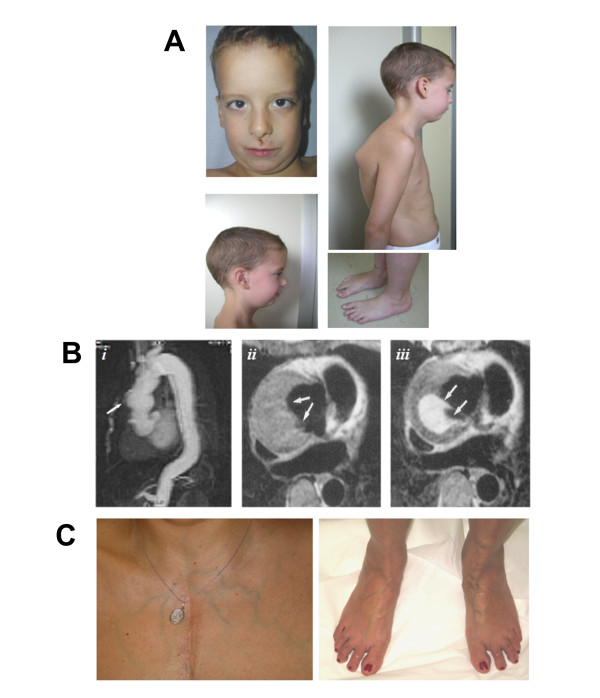
**Clinical and radiographic features of the two LDS Patients**. **A) **Phenotype of Patient 1, showing facial dysmorphisms: dolichocephaly, hypoplastic *alae nasi*, and micro/retrognathia; marfanoid *habitus*, muscular hypotrophy, mild thoracic scoliosis, and *pes planus*. **B) **Patient 2: Ascending aorta dilatation (***i***) and MRI axial T1 spin-echo images of the ascending aorta at the level of re-implanted coronary arteries, before (***ii***) and after (***iii***) gadolinium: a periaortic collection is visible (arrows), with contrast medium outside the aortic graft, due to detachment of re-implanted left coronary artery. **C) **Translucent skin with subcutaneous visible veins and flat feet in Patient 2.

Patient 1 was a seven years old male, born at term after uncomplicated pregnancy from non-consanguineous parents. At birth, weight and length were normal; he showed bilateral clubfoot and hand contractures characterized by camptodactyly and ulnar deviation. At the age of six years he showed: height 130.5 cm, arm span 129 cm, upper:lower segment *ratio *0.89, muscular hypotrophy; dolicocephaly, blue sclerae, hypoplastic *alae nasi*, microretrognathia, high-arched palate, bifid uvula; mild thoracic scoliosis and *pectus excavatum, pes planus*, joint hypermobility (Beighton score 8/9); velvety and translucent skin (Fig. 1A). Echocardiogram showed bicuspid aortic valve, patent foramen ovale, mild interventricular septal hypertrophy without outflow obstruction, mild mitral valve prolapse, aortic root diameter 23 mm and ascending aorta diameter 21 mm. Magnetic Resonance Angiography (MRA) displayed tortuosity and elongation of the basilar and internal carotid arteries with kinking and coiling of these last. Furthermore, the patient suffered from allergic asthma and atopic dermatitis.

Patient 2, a 48 years old woman, underwent several vascular surgery interventions: at the age of 26 years vascular prosthesis replacement of the ascending aorta with Bentall technique (composite graft and coronary arteries reimplant) for aortic aneurysm; eight years later, surgery for haematoma consequent to suture dehiscence at the level of the left coronary ostium; at the age of 43 years surgical repair of acute dissection starting from the distal anastomosis of the aortic graft extended to the abdominal aorta; at the age of 44 years, endovascular treatment with stent-graft of the descending aorta for increase of descending thoracic false lumen (Fig 1B). The patient showed: height 165 cm, velvety and translucent skin, and bilateral venous varicosities (Fig. 1C). The patient had a total of two pregnancies, the first uncomplicated, the second terminated at about 4 months of gestation because of the presence of the above mentioned aneurysm of the ascending aorta at high dissection risk. She had recurrent wrist dislocations in childhood. Proband's father suddenly died at the age of 33 years for unknown causes. The 26 years old proband's daughter did not present valvular, aortic or other anomalies during several echocardiographic evaluations. Her clinical history included a pituitary adenoma and she suffered from reactive hypoglycaemia and allergic rhinitis. Recurrent left patella dislocations were reported, without other signs of joint hypermobility. She showed mild myopia.

**Figure 2:**
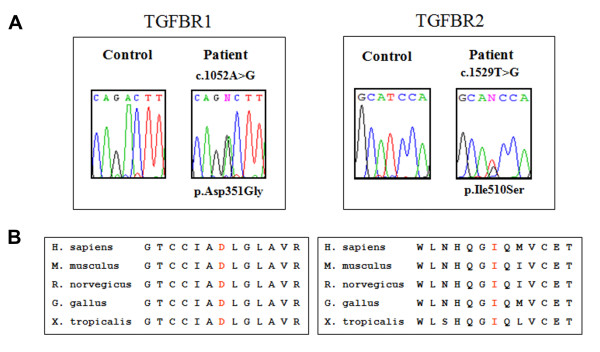
**Molecular characterization of the patients by *TGFBR1 *and *TGFBR2 *sequencing on genomic DNA, obtained from whole peripheral blood**. **A) **Sequence chromatogram of Patient 1, showing the position of the heterozygous c.1052A>G transition in *TGFBR1 *exon 6, leading to the p.Asp351Gly missense mutation, and chromatogram of Patient 2 showing the heterozygous c.1529T>G transversion in *TGFBR2 *exon 7, resulting in the p.Ile510Ser missense mutation. **B) **The p.Asp351Gly and p.Ile510Ser mutations substitute in LDS patients an aspartic acid and an isoleucine residues that are evolutionally conserved in *TGFBR1 *and *TGFBR2 *orthologues, respectively. Multiple sequence alignment was performed using CLUSTALW and PolyPhen.

In Patient 1, sequencing analysis of genomic DNA disclosed in exon 6 of the *TGFBR1 *gene the c.1052A>G transition, leading to the substitution of an aspartic acid with a glycine in position 351 of the aminoacidic sequence (p.Asp351Gly) (Fig. 2A). This novel missense mutation was not detected in the proband's parents, suggesting that it arose as a *de novo *event. In Patient 2 and in her daughter, *TGFBR2 *genotyping disclosed the c.1529T>G transversion in exon 7, resulting in the p.Ile510Ser missense mutation (Fig. 2A). The proband's father was likely affected with LDS, as he suddenly died at a young age. Both missense mutations here identified were not detected in 250 chromosomes of control Italian blood donors, and substituted residues in the kinase domain of the proteins, which is highly conserved in the homologous of the receptor (Fig. 2B). Furthermore, in silico prediction algorithms, polymorphism phenotyping (PolyPhen) and sorting intolerant from tolerant (SIFT) [3], characterized both the p.Asp351Gly and p.Ile510Ser substitutions as damaging and affecting protein function, respectively.

Patient 1 showed suggestive signs (*i.e*., marfanoid *habitus *without fulfilled Ghent criteria for MFS, facial, skeletal, cutaneous and cardiovascular involvement) consistent with LDSI phenotype. Patient 2 showed the absence of cardinal features of LDS, such as arterial tortuosity, bifid uvula, hypertelorism, micrognathia, and joint hypermobility. These observations, associated with the presence of translucent skin and of marked venous varicosities, are consistent with a LDSII phenotype. In particular, Patient 2 resembled vEDS for skin presentation and joint involvement, that generally is less evident in vEDS than in other EDS types and in LDS patients [2]. However, the straight involvement of the thoracic aorta of Patient 2 is more suggestive of an LDS rather than a vEDS phenotype, since in vEDS the medium-sized arteries are more frequently affected than aorta and great vessels [14]. So far, 21 LDSII patients were reported, some of which did not show aortic root dilatation, arterial tortuosity, or both [2,13,15]. Remarkably, the daughter of Patient 2 showed at 26 years few disease signs, supporting previous observations in LDS intrafamiliar variability.

In the *TGFBR*s-related disorders no genotype-phenotype correlation has been drawn, and the prediction of the clinical effects of different *TGFBR*s mutations remains difficult to envisage [1,9]. All the clinical entities caused by mutations in *TGFBR*s genes should be considered a phenotypic *continuum *of the same disease, ranging from isolated thoracic aneurysm to syndromic vascular rupture, and LDS should be considered a complex syndrome characterized by full penetrance (with very rare exceptions) [2], marked variable intra- and inter-familiar expressivity, allelic and genetic heterogeneity, pleiotropic effects, and high incidence of *de novo *events. On the other hand, LDS shows marked overlapping signs with other connective tissue disorders, due to different gene defects and characterized by vascular fragility, such as MFS (MIM 154700), vEDS (MIM 130050), and arterial tortuosity syndrome (ATS, MIM 208050), due to mutations in *SLC2A10 *gene and characterized by facial features, aneurysm ruptures, great vessels tortuosity, and pulmonary artery stenosis [1,2,6,13,16]. The allelic heterogeneity, as well as the clinical overlapping, could be related to the underlying pathophysiology of LDS, MFS, and MFS2 syndromes, and probably ATS, sharing alterations in similar molecular pathways [1,2,16]. Indeed, in these disorders, aortic aneurysm is associated with altered collagens deposition, elastic fibers disarray, and increased TGFbeta signalling, and is prevented by TGFbeta antagonists in the murine model [1,2,5,16,17].

## Conclusion

We report two novel mutations in the *TGFBR1 *and *TGFBR2 *genes in two patients affected with LDS and showing marked phenotypic variability. Due to the difficulties in the clinical approach to a TGFBR-related disease, among patients with vascular involvement, with or without aortic root dilatation and LDS cardinal features, genotyping is mandatory to clarify the diagnosis, to assess the management and the prognosis, and to satisfy counselling issues.

Further genotype-phenotype studies in these connective tissue disorders, together with the comprehension of the role of the genetic factors responsible of the arterial wall defects, will provide new insights in vascular diseases. The pathogenesis, as well as the phenotype of LDS, could be influenced by other genetic factors, either *TGFBR*s intragenic functional polymorphisms and different modifier genes, encoding for TGFbeta pathway proteins, or components of the vascular extracellular matrix.

## Consent

Written informed consent was obtained from patients for publication of this case report and accompanying images. A copy of the written consent is available for review by the Editor-in-Chief of this journal.

## List of abbreviation used

LDS: Loeys-Dietz syndrome; LDSI: LDS type I; LDSII: LDS type II; TGFBR1: transforming growth factor beta receptor 1; TGFBR2: transforming growth factor beta receptor 2; TGF: transforming growth factor; MFS: Marfan syndrome; vEDS: vascular Ehlers-Danlos syndrome; FS: Furlong syndrome; TAAD: familial thoracic aortic aneurysms and dissection: MRA: Magnetic Resonance Angiography.

## Competing interests

The authors declare that they have no competing interests.

## Authors' contributions

MC conceived the study. BD and MR carried out the molecular analysis, researched the literature reviewed and prepared the manuscript. AW, RF, BD, and PGCP diagnosed the patients and drafted the initial manuscript. AW, RF, NZ, and PGC-P contributed to the discussion section. SB and MC edited and coordinated the manuscript. All authors read and approved the final manuscript.
